# Exploring Fear of Falling Related Activity Avoidance among Postmenopausal Women

**DOI:** 10.3390/ijerph18084042

**Published:** 2021-04-12

**Authors:** Sukhee Ahn, Rhayun Song

**Affiliations:** College of Nursing, Chungnam National University, Daejeon 35015, Korea; sukheeahn@cnu.ac.kr

**Keywords:** postmenopause, accidental falls, activities of daily living, fall efficacy

## Abstract

Fear of falling was associated with activity avoidance and subsequent reduction of functioning capacity in postmenopausal women. This study aimed to determine the influencing factors for fear-of-falling related activity avoidance in Korean postmenopausal women. The sample of 687 postmenopausal women living in six urban areas was recruited using stratified convenience sampling for the original survey. A secondary analysis was applied to survey data from 541 postmenopausal women aged 50–64 years who experienced some degree of fear of falling. A structured questionnaire was administered to measure fear of falling, fall efficacy, and activity avoidance. Among 541 postmenopausal women with a mean age of 56 years who perceived at least some fear of falling, 15% (*n* = 81) reported they avoided performing some, not all, activities of daily living due to fear of falling. Fear of falling was significantly associated with the level of activity avoidance (*χ*^2^ = 16.94, *p* < 0.001). In multivariate analyses, fear of falling and fall efficacy were significant predictors of activity avoidance in postmenopausal women after adjusting for age, education level, and chronic disease. Fear of falling and fall efficacy contributed independently to explain activity avoidance in postmenopausal women. It is important to identify fear of falling and activity avoidance as the focus of public health in order to prevent the vicious cycle of future falls.

## 1. Introduction

Fear of falling, which is also known as post-fall syndrome, may present in fallers as well as in individuals who have not yet experienced a fall [1,2]. Since the fear of falling is very common in both fallers and non-fallers, this could be associated with falls via shared risk factors [3]. It has been hypothesized that fear of falling contributes to worse balance and decreased functional ability that consequently leads to activity restriction, which together plays an important role in causing future falls [4].

A previous analysis of national survey data found that fear of falling was the most important factor in predicting activity avoidance among older adults [5,6]. Experiencing a fall could result in a fear of falling and a consequent avoidance of performing activities of daily living, and physical inactivity would impair the physical condition and reduce muscle strength, in turn increasing the risk of falling [7].

Interventions for fall prevention should focus on managing the fear of falling since this fear-initiated vicious cycle is a key to explaining the functional decline in both older fallers and non-fallers [8].

Previous fall-related research has mainly focused on falls in older adults [5,6]. However, postmenopausal women younger than 65 years also potentially have an increased risk of falling [9,10]. Postmenopausal women have a high risk of osteoporosis and fall-related fractures due to a dramatic decrease in estrogen and the subsequent decreases in lean body mass and bone loss [9]. A study involving a Korean population found that 19% of young postmenopausal women aged between 50 and 65 years had fallen at least once during the previous year, and 39% of those who fell suffered fractures [11]. An epidemiological study that analyzed a longitudinal database of 978 women in mean age 65.9 years also found that 33.5% of the women had fallen at least once in past 12 months [10]. Fear of falling has been shown to be a predictor of falls in postmenopausal women [12]. Those authors concluded that the presence of FoF related activity avoidance in young postmenopausal women may be indicative of the early reduction of functioning capacity, subsequently leading to the vicious cycle of falling in later life.

Current literature on fear of falling and FoF related avoidance of activity indicates some gaps for further studies. First, factors independently related to fear of falling and particularly for avoidance of activity due to fear of falling are understudied in postmenopausal women. Second, previous studies [12,13,14,15] suggested socio-demographics (age, education level, and living status) and/or health-related variables (perceived health, comorbidity, history of falling, etc.) as influencing factors on fear of falling, yet hardly any study incorporated an extensive range of potential correlates of fear of falling with avoidance of activity and fall efficacy. Moreover, little is known about Korean populations with fear of falling and avoidance of activity, along with duration since menopause, anxiety, higher body mass index, history of falls, older age, lower education, and comorbidity as potential influencing factors [12,13,15]. Furthermore, it is relevant and very important to differentiate between mild and severe levels of fear of falling and activity avoidance. Those people with severe fear of falling and subsequent avoidance of activity would be the target population relevant for interventions. Such knowledge of these factors may help to specify the contents of interventions for them.

While activity avoidance due to a fear of falling may play an important role in increasing the risk of falling in this population, little is known about the related risk factors for activity avoidance among postmenopausal women younger than 65 years. Since these women are close to becoming part of the elderly population and consequently more vulnerable to falls and subsequent fractures, more attention should be given to fall prevention in this population.

The purposes of the study were to determine the prevalence of FoF related activity avoidance in postmenopausal women, and identify demographic, health-related, and psychosocial factors that are predictive factors for activity avoidance in this population.

## 2. Methods

### 2.1. Design

The secondary data analysis was conducted with the survey data from 541 postmenopausal women. The data were from a larger study with a cross-sectional design that explored the risk factors for osteoporosis and falls in postmenopausal women aged between 50 and 64 years [11].

### 2.2. Participants

The sample of 687 postmenopausal women younger than 65 years living in six urban areas in South Korea were recruited using stratified convenience sampling for the original study [11]. The inclusion criteria was (1) women between 50 to 64 years old, (2) being postmenopausal state longer than 12 months without having menstrual period, (3) being able to perform activities of daily life, and (4) being able to communicate and agree to participate in the study with signed consent form. As an additional inclusion criteria for the present study to screen the participants who experienced fear of falling using the single-item fear of falling question [16]. The total of 541 postmenopausal women who perceived a mild or greater fear of falling were included in the present secondary data analysis. Considering the focus of the present study on FoF related activity avoidance, those who reported no fear of falling (*n* = 146, 21.3%) were excluded. The power analysis was performed to confirm the sample size for the secondary analysis with logistic regression, the required sample size was 172 cases with 10 predictors, effect size 0.10, and power 0.80, and the obtained data of 541 was enough for the analysis.

### 2.3. Measurements

The influencing factors for FoF related activity avoidance included demographic characteristics (age, education level, and living alone or with others), health-related variables (perceived health, presence of chronic disease, osteoporosis diagnosis, a history of falling, and fall-related fracture during the previous year), and psychosocial variables (fear of falling and fall efficacy). This self-reported structured questionnaire took about 20 min to complete.

Fear of falling: Fear of falling was measured using a single question (How serious is your fear of falling?) that was scored in a four-point Likert-type format from 0 to 3 (none, mild, moderate, and severe, respectively) [16]. This question was used to screen participants who perceived at least some degree of fear of falling when performing activities of daily living, with the answers also recoded into a dummy variable with values of 0 (mild) and 1 (moderate or severe) for the regression analysis. 

Fall efficacy: Fall efficacy is the belief regarding their certainty about preventing or avoiding falls, and was measured using a seven-item fall efficacy scale in a five-point format from 1 (not sure at all) to 5 (very sure). The fall efficacy scale was developed and validated for original study with face validity and content validity in Korean postmenopausal women [11]. The internal consistency of this scale was also reported with a Cronbach’s alpha of 0.90 [17].

FoF related activity avoidance: FoF related activity avoidance was assessed using an activity avoidance scale modified based on a fear-of-falling questionnaire [16]. The scale consists of 11 items scored using a four-point Likert-type format (never, rarely, often, and always) to assess how frequently postmenopausal women would avoid performing activities of daily living due to a fear of falling. FoF related activity avoidance scale was developed and validated by Tideiksaar [16]. It was translated into Korean and validated with face validity and content validity in Korean postmenopausal women [17]. Reliability of the Korean version of the scale was reported as 0.91 [17].

### 2.4. Ethical Considerations

The institutional review board (IRB) approved the original survey (CNU #11-02), which was conducted according to the guidelines of the Declaration of Helsinki. Informed consent was obtained from all subjects involved in the original study. The present study with the secondary use of existing data excluding identifiable information on the selected subjects was exempt from IRB review and approval.

### 2.5. Statistical Methods

Analyses were conducted using SPSS for Windows V. 24. A significance level of less than 0.05 was considered statistically significant. Frequency and descriptive statistics were used to quantify the fear of falling and FoF related activity avoidance in postmenopausal women. Fall efficacy as a continuous variable was assessed for normality with skewness (0.002/0.106) and kurtosis (0.138/0.211) prior to the regression analysis. Chi-square and *t*-tests were performed to compare the demographic, health-related, and psychosocial variables between groups categorized based on the level of activity avoidance. Multiple logistic regression analysis was conducted to identify the significant variables for predicting FoF related activity avoidance. The scores of FoF related activity avoidance were dummy coded into 0 (never or rarely) and 1 (often or always) as a dependent variable. The demographic, health-related, and psychosocial variables with significant univariate associations were included in the analysis as predictive factors for the dependent variable. Demographic factors were entered first to control its effect as non-modifiable factors. Then health-related factors were subsequently entered in Model 2 to see the effects on the outcome as related risk factors. Finally, fear of falling and fall efficacy as main study variables were entered in Model 3. Odds ratios (ORs) and corresponding 95% confidence intervals (CIs) were estimated.

## 3. Results

### 3.1. Fear of Falling, Fall Efficacy, and Activity Avoidance

Among 541 postmenopausal women who perceived at least some fear of falling, 15% (*n* = 81) avoided some of the activities of daily living. The postmenopausal women never or rarely avoided activities such as getting on/off the toilet (96.3%) or walking alone indoors (97.5%) or outdoors (93.8%). The activities that most women often or always avoided due to a fear of falling were descending stairs/curbs (30%), climbing stairs/curbs (25.4%), and reaching up into closets/cabinets (23.7%) (Table 1).

### 3.2. General Characteristics of the Subjects According to Activity Avoidance Groups

Table 2 lists the demographic and health-related characteristics of the participants according to the level of FoF related activity avoidance, categorizing 85% (*n* = 460) into the ‘rarely’ group (never or rarely avoid activities) and 15% (*n* = 81) to the ‘always’ group (often or always avoid activities). The participants were 56 years old on average, and more than half of them had an education level of high-school graduate or higher. About 15% of the women rated their health as poor to very poor, and only 5.4% of them were living alone. Age and education level differed significantly according to the level of activity avoidance.

For health-related factors, 23.4% of the women had an osteoporosis diagnosis, and about half of them had a chronic disease such as hypertension or osteoarthritis. More than 20% of the women reported falling at least once during the past 12 months, and 7.9% had experienced a fall related fracture. Chronic disease and an osteoporosis diagnosis were significantly associated with the level of FoF related activity avoidance in the postmenopausal women.

For psychosocial factors, about 20% of the women had moderate to severe fear of falling, while 80% perceived mild fear of falling. Fall efficacy score was different by level of FoF related activity avoidance (Table 2).

### 3.3. Fall Efficacy and Fear of Falling According to FoF Related Activity Avoidance

Table 3 presents the associations of fear of falling and fall efficacy with the level of FoF related activity avoidance. The fear of falling was significantly associated with the level of activity avoidance (*χ*^2^ = 16.94, *p* < 0.001). While 83.3% of those with mild fear of falling and 4.8% of those with severe fear of falling never or rarely avoided performing activities of daily living, 65.4% and 14.8% of them, respectively, avoided some activities often or always. The total score for fall efficacy was 26.32 ± 4.52 (ranging from 9 to 35), indicating a moderate level of confidence in their ability to prevent falling. The score for fall efficacy differed significantly with the level of activity avoidance in the postmenopausal women (*t* = 3.65, *p* < 0.001) (Table 3).

### 3.4. Explanatory Factors for FoF Related Activity Avoidance

Study variables showing significant univariate associations with FoF related activity avoidance were entered in the order of demographic (age and education level), health-related (chronic disease and osteoporosis diagnosis), and psychosocial (fear of falling and fall efficacy) variables. Perceived health and a history of falling that were supported as predictive variables in previous studies were also included in the model.

Demographic variables of age and education level were entered first into model 1, which revealed that older age (OR = 1.07) and lower education level (OR = 2.00) were significantly associated with activity avoidance. Health-related variables were then entered into model 2, which resulted in a lower education level remaining a significant factor (OR = 1.84). When psychosocial variables were added to model 3, fear of falling and fall efficacy were significant factors even after adjusting for demographic and health-related variables. Women who had a severe fear of falling were more than twice as likely to avoid activities (OR = 2.09, 95% CI = 1.17–3.73), while those with greater fall efficacy were 0.9 times as likely to avoid activities (OR = 0.89; 95% CI = 0.84–0.94) (Table 4).

## 4. Discussion

This study found that among 541 postmenopausal women with a mean age of 56 years who perceived at least some fear of falling, 15% (*n* = 81) reported they avoided performing some, not all, activities of daily living due to fear of falling. In contrast, 10.1% of somewhat older women (with a mean age of 64 years) were previously found to perform FoF related activity avoidance (Martin et al., 2005). The most frequently avoided activities were descending or climbing stairs followed by reaching up into closets. A history of falling has been associated with fear of falling in older adults [3]. However, we found no significant association with a history of falling during the previous year, while being old and having a low education level and chronic disease were significantly associated with the level of activity avoidance. Previous studies have also supported that fear of falling is commonly present in postmenopausal and older women who have either fallen or never fallen previously [12]. No significant association we found in the study could be due to the tendency that those women with fear of falling would reduce the chance to go out or doing outdoor activities much less than those with less fear of falling.

As expected, the fear of falling was significantly associated with the level of activity avoidance—the degree of fear of falling seems to be an important barrier to postmenopausal women engaging in the activities of daily living. A previous study also suggested that the degree to which individuals are afraid of falling when performing activities of daily living is positively associated with the level of activity avoidance, even after adjusting for the history of falling [18].

The association between fear of falling and activity avoidance in postmenopausal women has become a major public health concern among health-care professionals [13,14,17]. Fear of falling along with FoF related activity avoidance could limit physical activity in this population, leading to adverse consequences—such as functional decline, restriction of social participation, decreased quality of life, and increased risk of falling—especially since they are close to becoming part of older adults population [3,4]. Health-promotion strategies should be focused on the fear of falling and associated activity avoidance in the middle-aged population in order to prevent subsequent falls among older adults.

Our multiple logistic regression analysis revealed that aging and lower education level were significantly associated with FoF related activity avoidance in postmenopausal women. However, fear of falling and fall efficacy were independent factors explaining FoF related activity avoidance even after adjusting for demographic and health-related factors. Fall efficacy has previously been recognized as a significant predictor of future falls in postmenopausal women [19]. Therefore, these modifiable psychosocial variables could be a useful target when developing effective fall prevention strategies in this population.

The findings of this study should be interpreted while considering the following limitations. The present secondary analysis quantified the fear of falling using a single question about the level of concern relating to falls during the activities of daily living. A single question to assess the presence of fear of falling has also been used in previous studies with good test–retest reliability [20], and shown to be correlated with scores on the Tinetti Falls Efficacy Scale and with physical activity [4,21]. However, we need to consider potential bias in the accuracy of categorizing the level of fear of falling using a single question. Another limitation of this study is related to inherent features of a secondary data analysis. Our original study [11] used stratified convenience sampling of six urban areas in South Korea rather than recruiting representative samples, and the resulting sample mainly comprised women who were married (more than 90%) and highly educated (79% were high-school or college graduates). Therefore, while we controlled the effects of demographic variables to explain FoF related activity avoidance among postmenopausal women, caution is necessary when generalizing our findings.

The present findings may provide health-care professionals with insight into how to assess fear of falling and FoF related activity avoidance among postmenopausal women. The health-care professionals should consider several points when constructing fall prevention strategies for postmenopausal women. First, it is important to assess the level of FoF related activity avoidance in this population along with pre-existing health concerns such as depression or cognitive impairment that may initiate a vicious cycle of accidental falls. Second, it is important to identify the common factors associated with a high degree of fear of falling and FoF related activity avoidance, since such knowledge may help in determining the optimal contents, approach, and length of interventions for this population. Third, intervention studies that apply cognitive and behavioral approaches are warranted to promote fall prevention behaviors and to evaluate fall-prevention-related outcomes by focusing on the fear of falling and fall efficacy in postmenopausal women. The potential moderating effect of fear of falling on the relationship between fall efficacy and activity avoidance are worth exploring further.

## 5. Conclusions

We found that fear of falling was significantly associated with the level of activity avoidance in postmenopausal women. Both fear of falling and fall efficacy were independent explanatory factors of FoF related activity avoidance in this population. It is important to identify individuals at risk of fear-of-falling and related activity avoidance, since they can receive interventions aimed at reducing fear of falling and increasing their activities of daily living, consequently preventing the vicious cycle of future falls.

## Figures and Tables

**Table 1:** Distribution of fear-related activity avoidance (*N* = 541).

Activities of Daily Living	Fear of Falling Related Activity Avoidance			
	Never (*n* = 346, 64%)	Rarely (*n* = 114, 21%)	Often (*n* = 70, 13%)	Always (*n* = 11, 2%)
1. Walking alone outdoors	386 (71.3)	121 (22.4)	30 (5.5)	4 (0.7)
2. Walking alone indoors	426 (78.7)	101 (18.7)	10 (1.8)	4 (0.7)
3. Getting on/off the toilet	427 (78.9)	94 (17.4)	19 (3.5)	1 (0.2)
4. Getting in/out of the bathtub/shower	370 (68.4)	123 (22.7)	41 (7.6)	7 (1.3)
5. Taking a bath/shower	394 (72.8)	122 (22.6)	21 (3.9)	4 (0.7)
6. Getting in/out of chairs	385 (71.2)	126 (23.3)	30 (5.5)	0 (0.0)
7. Getting on/off the bed	386 (71.3)	126 (23.3)	26 (4.8)	3 (0.6)
8. Reaching up into closets/cabinets	261 (48.2)	152 (28.1)	108 (20.0)	20 (3.7)
9. Bending down to place/retrieve objects	305 (56.4)	160 (29.6)	68 (12.6)	8 (1.5)
10. Climbing stairs/curbs	274 (50.6)	130 (24.0)	114 (21.1)	23 (4.3)
11. Descending stairs/curbs	245 (45.3)	134 (24.8)	128 (23.7)	34 (6.3)

Data are *n* (%) values.

**Table 2:** General characteristics of the participants according to degree of fear-related activity avoidance (*N* = 541).

		Fear-Related Activity Avoidance		*χ* ^2^	*p*
	Category	Rarely (*n* = 460)	Always (*n* = 81)		
*Demographic factors*					
Age (years)	50–54	240 (52.2)	29 (35.8)	15.37	<0.001
	55–59	136 (29.6)	22 (27.2)		
	60–64	84 (18.3)	30 (37.0)		
Education level	Middle school	78 (17.0)	28 (34.6)	16.50	<0.001
	High school	200 (43.4)	35 (43.2)		
	College or higher	182 (39.6)	18 (22.2)		
Living status	Living alone	23 (5.0)	6 (7.4)	0.78	0.375
	Living with others	437 (95.0)	75 (92.6)		
*Health-related factors*					
Perceived health	Poor/very poor	64 (13.9)	18 (22.2)	3.69	0.054
	Good/excellent	396 (86.1)	63 (77.8)		
Chronic disease	Yes	209 (45.4)	53 (65.4)	11.02	0.001
	No	251 (54.6)	28 (34.6)		
Osteoporosis	Yes	98 (21.3)	29 (35.8)	8.05	0.045
	No	362 (78.7)	52 (64.2)		
History of falling	Yes	88 (19.1)	23 (28.4)	3.62	0.057
	No	372 (80.9)	58 (71.6)		
Fall-related fracture	Yes	34 (7.4)	9 (11.1)	1.30	0.254
	No	426 (92.6)	72 (88.9)		
*Psychosocial factors*					
Fear of falling	Mild	383 (83.3)	53 (65.4)	0.167 ^+^	<0.001
	Moderate	55 (12.0)	16 (19.8)		
	Severe	22 (4.8)	12 (14.8)		
Fall efficacy		26.61 ± 4.46	24.61 ± 4.51	−0.153 ^++^	<0.001

Data are *n* (%) or mean ± SD values. ^+^ indicates statistical value of Spearman rho, ^++^ indicates r.

**Table 3:** Fall efficacy and fear of falling according to fear-related activity avoidance (*N* = 541).

Variable		Fear of Falling Related Activity Avoidance			
		Rarely (*n* = 460)	Always (*n* = 81)		
Degree of fear of falling				*χ* ^2^	*p*
Mild	436 (80.5)	383 (83.3)	53 (65.4)	16.94	<0.001
Moderate	71 (13.1)	55 (12.0)	16 (19.8)		
Severe	34 (6.2)	22 (4.8)	12 (14.8)		
				*t*	*p*
Fall efficacy		26.61 ± 4.46	24.61 ± 4.51	3.65	<0.001

Data are *n* (%) or mean ± SD values.

**Table 4:** Explanatory factors for fear-related activity avoidance (*N* = 541).

	Model 1	Model 2	Model 3		
	OR *p*	OR *p*	OR	95% CI	*p*
*Demographic Factors*					
Age	1.07 0.008	1.05 0.084	1.05	(0.98–1.11)	0.112
Low education level (ref: at least high school)	2.00 0.014	1.84 0.035	1.68	(0.94–3.00)	0.079
*Health-related factors*					
Poor perceived health (ref: good to excellent)		1.39 0.307	1.01	(0.51–1.99)	0.965
Chronic disease (ref: no)		1.60 0.092	1.38	(0.91–2.09)	0.127
Osteoporosis diagnosis (ref: no)		1.35 0.297	1.31	(0.73–2.36)	0.358
History of falling (ref: no)		1.55 0.200	1.57	(0.78–3.14)	0.426
*Psychosocial factors*					
Fear of falling (ref: mild)			2.09	(1.17–3.73)	0.012
Fall efficacy			0.89	(0.84–0.94)	<0.001

OR, odds ratio; CI, confidence interval.

## Data Availability

Not applicable.
